# Supplementary material for: Unveiling the Bioactive Potential of the Invasive Jellyfish Phyllorhiza punctata Through Integrative Transcriptomic and Proteomic Analyses
Source: Biomolecules. 2025 Aug 4;15(8):1121. doi: 10.3390/biom15081121 (PMC12383608; doi:10.3390/biom15081121)
Supplement: Supplementary file 1 [file biomolecules-15-01121-s001.zip › Suplementary Material/FileS3/PEPFOLD4/XP_065071755_4832/clusters.html]

 PEP-FOLD clusters


Download archive of all models  here   

|  |  |  |
| --- | --- | --- |
| # | file | sOPEP |
| # cluster 1 - Size: 36 |  |  |
|  | XP\_065071755\_4832-model1 | -9.65114 |
|  | XP\_065071755\_4832-model1.1 | -9.65114 |
|  | XP\_065071755\_4832-model1.2 | -9.65114 |
|  | XP\_065071755\_4832-model1.3 | -9.65114 |
|  | XP\_065071755\_4832-model1.4 | -9.65114 |
|  | XP\_065071755\_4832-model1.5 | -9.65114 |
|  | XP\_065071755\_4832-model1.6 | -9.65114 |
|  | XP\_065071755\_4832-model1.7 | -9.65114 |
|  | XP\_065071755\_4832-model1.8 | -9.65114 |
|  | XP\_065071755\_4832-model1.9 | -9.65114 |
|  | XP\_065071755\_4832-model1.10 | -9.65114 |
|  | XP\_065071755\_4832-model1.11 | -9.65114 |
|  | XP\_065071755\_4832-model1.12 | -9.65114 |
|  | XP\_065071755\_4832-model1.13 | -9.65114 |
|  | XP\_065071755\_4832-model1.14 | -9.65114 |
|  | XP\_065071755\_4832-model1.15 | -9.65114 |
|  | XP\_065071755\_4832-model1.16 | -9.65114 |
|  | XP\_065071755\_4832-model1.17 | -9.65114 |
|  | XP\_065071755\_4832-model1.18 | -9.65114 |
|  | XP\_065071755\_4832-model1.19 | -9.65114 |
|  | XP\_065071755\_4832-model1.20 | -9.65114 |
|  | XP\_065071755\_4832-model1.21 | -9.65114 |
|  | XP\_065071755\_4832-model1.22 | -9.65114 |
|  | XP\_065071755\_4832-model1.23 | -9.65114 |
|  | XP\_065071755\_4832-model1.24 | -9.65114 |
|  | XP\_065071755\_4832-model1.25 | -9.65114 |
|  | XP\_065071755\_4832-model1.26 | -9.65114 |
|  | XP\_065071755\_4832-model1.27 | -9.65114 |
|  | XP\_065071755\_4832-model1.28 | -9.65114 |
|  | XP\_065071755\_4832-model1.29 | -9.65114 |
|  | XP\_065071755\_4832-model1.30 | -9.65114 |
|  | XP\_065071755\_4832-model1.31 | -9.53923 |
|  | XP\_065071755\_4832-model1.32 | -9.18632 |
|  | XP\_065071755\_4832-model1.33 | -9.1458 |
|  | XP\_065071755\_4832-model1.34 | -6.0221 |
|  | XP\_065071755\_4832-model1.35 | -5.69924 |
| # cluster 2 - Size: 39 |  |  |
|  | XP\_065071755\_4832-model2 | -5.04545 |
|  | XP\_065071755\_4832-model2.1 | -5.01432 |
|  | XP\_065071755\_4832-model2.2 | -4.99729 |
|  | XP\_065071755\_4832-model2.3 | -4.99729 |
|  | XP\_065071755\_4832-model2.4 | -4.99442 |
|  | XP\_065071755\_4832-model2.5 | -4.95983 |
|  | XP\_065071755\_4832-model2.6 | -4.95983 |
|  | XP\_065071755\_4832-model2.7 | -4.95983 |
|  | XP\_065071755\_4832-model2.8 | -4.95349 |
|  | XP\_065071755\_4832-model2.9 | -4.93294 |
|  | XP\_065071755\_4832-model2.10 | -4.93294 |
|  | XP\_065071755\_4832-model2.11 | -4.93294 |
|  | XP\_065071755\_4832-model2.12 | -4.87319 |
|  | XP\_065071755\_4832-model2.13 | -4.85362 |
|  | XP\_065071755\_4832-model2.14 | -4.85362 |
|  | XP\_065071755\_4832-model2.15 | -4.84681 |
|  | XP\_065071755\_4832-model2.16 | -4.84446 |
|  | XP\_065071755\_4832-model2.17 | -4.80497 |
|  | XP\_065071755\_4832-model2.18 | -4.78367 |
|  | XP\_065071755\_4832-model2.19 | -4.74875 |
|  | XP\_065071755\_4832-model2.20 | -4.74875 |
|  | XP\_065071755\_4832-model2.21 | -4.74584 |
|  | XP\_065071755\_4832-model2.22 | -4.74584 |
|  | XP\_065071755\_4832-model2.23 | -4.741 |
|  | XP\_065071755\_4832-model2.24 | -4.741 |
|  | XP\_065071755\_4832-model2.25 | -4.74055 |
|  | XP\_065071755\_4832-model2.26 | -4.73683 |
|  | XP\_065071755\_4832-model2.27 | -4.71956 |
|  | XP\_065071755\_4832-model2.28 | -4.71073 |
|  | XP\_065071755\_4832-model2.29 | -4.68231 |
|  | XP\_065071755\_4832-model2.30 | -4.68231 |
|  | XP\_065071755\_4832-model2.31 | -4.68142 |
|  | XP\_065071755\_4832-model2.32 | -4.68142 |
|  | XP\_065071755\_4832-model2.33 | -4.61087 |
|  | XP\_065071755\_4832-model2.34 | -4.61087 |
|  | XP\_065071755\_4832-model2.35 | -4.58851 |
|  | XP\_065071755\_4832-model2.36 | -4.53799 |
|  | XP\_065071755\_4832-model2.37 | -4.47361 |
|  | XP\_065071755\_4832-model2.38 | -4.30915 |
| # cluster 3 - Size: 10 |  |  |
|  | XP\_065071755\_4832-model3 | -5.00667 |
|  | XP\_065071755\_4832-model3.1 | -5.00667 |
|  | XP\_065071755\_4832-model3.2 | -4.96994 |
|  | XP\_065071755\_4832-model3.3 | -4.92589 |
|  | XP\_065071755\_4832-model3.4 | -4.90534 |
|  | XP\_065071755\_4832-model3.5 | -4.70406 |
|  | XP\_065071755\_4832-model3.6 | -4.51755 |
|  | XP\_065071755\_4832-model3.7 | -4.51132 |
|  | XP\_065071755\_4832-model3.8 | -4.45304 |
|  | XP\_065071755\_4832-model3.9 | -4.44168 |
| # cluster 4 - Size: 3 |  |  |
|  | XP\_065071755\_4832-model4 | -4.99909 |
|  | XP\_065071755\_4832-model4.1 | -4.52249 |
|  | XP\_065071755\_4832-model4.2 | -4.46703 |
| # cluster 5 - Size: 2 |  |  |
|  | XP\_065071755\_4832-model5 | -4.73216 |
|  | XP\_065071755\_4832-model5.1 | -4.32778 |
| # cluster 6 - Size: 8 |  |  |
|  | XP\_065071755\_4832-model6 | -4.29463 |
|  | XP\_065071755\_4832-model6.1 | -4.29463 |
|  | XP\_065071755\_4832-model6.2 | -4.29463 |
|  | XP\_065071755\_4832-model6.3 | -4.28271 |
|  | XP\_065071755\_4832-model6.4 | -4.26117 |
|  | XP\_065071755\_4832-model6.5 | -4.24632 |
|  | XP\_065071755\_4832-model6.6 | -4.23337 |
|  | XP\_065071755\_4832-model6.7 | -4.20091 |
| # cluster 7 - Size: 2 |  |  |
|  | XP\_065071755\_4832-model7 | -4.22333 |
|  | XP\_065071755\_4832-model7.1 | -4.22333 |
|  |  |  |
